# Geo-Located Tweets. Enhancing Mobility Maps and Capturing Cross-Border Movement

**DOI:** 10.1371/journal.pone.0129202

**Published:** 2015-06-18

**Authors:** Justine I. Blanford, Zhuojie Huang, Alexander Savelyev, Alan M. MacEachren

**Affiliations:** 1 Department of Geography, GeoVISTA Center, Penn State University, 320 Walker, University Park, Pennsylvania, 16802, United States of America; 2 Centre for Infectious Disease Dynamics, Penn State University, Millenium Science Complex, University Park, Pennsylvania, 16802, United States of America; University of Namur, BELGIUM

## Abstract

Capturing human movement patterns across political borders is difficult and this difficulty highlights the need to investigate alternative data streams. With the advent of smart phones and the ability to attach accurate coordinates to Twitter messages, users leave a geographic digital footprint of their movement when posting tweets. In this study we analyzed 10 months of geo-located tweets for Kenya and were able to capture movement of people at different temporal (daily to periodic) and spatial (local, national to international) scales. We were also able to capture both long and short distances travelled, highlighting regional connections and cross-border movement between Kenya and the surrounding countries. The findings from this study has broad implications for studying movement patterns and mapping inter/intra-region movement dynamics.

## Introduction

Despite the importance of capturing human mobility, reliable data on movement is often scarce, imprecise, and out of date. Therefore efforts needed to aid in the planning and allocation of resources are often hampered by lack of accurate and verifiable mobility data, making it difficult to identify movement trends and patterns [1]. Many data streams have been used to capture aspects of human movement, and each has limits of currency, spatial and temporal precision, accuracy, geographic coverage, and/or representativeness of population of interest. Census data, for example, is widely available and commonly used for analyzing long-term net movement flows at broad spatial scales [2]. But census data are usually aggregated to political districts (thus are imprecise) and collected on a decennial cycle (thus are often out of date) [3]. Spatially referenced data from cell phones [4–6], circulation of the US dollar bill [7–9], air traffic data [10, 11], sequential night-time imagery [12], transportation data [13] and news articles [14] have also been used to estimate movement and serve as proxies for capturing mobility at various temporal scales. Airline traffic data, although good at showing connectivity between geographic locations and potential net flows, are restrictive since they only capture air transportation and long-range spatial patterns between specific origin-destination locations by individuals who can afford to fly. Data about circulation of US dollar bills provides mobility patterns across different scales but, currency movement is only an indirect proxy for human movement and the available data reflect only a small number of volunteer contributors [8, 9]. Night-time imagery is useful for capturing periodic movement such as seasonal variation [12] as are news articles through the reporting of an event [14]. Although cell phone call record data are useful for capturing population distribution [15] and fine scale spatial movement [16], the analysis of these data are usually aggregated to the nearest cell phone tower, can be difficult to obtain and are restricted in their use and reuse due to both proprietary and privacy issues [17]. Furthermore due to cell phone networks and coverage, these data are generally restricted to country-level, making cross-border human movement difficult to capture. Therefore, more accessible data through which human movement can be analyzed across different spatial and temporal scales and through different transport systems is needed [9].

With the arrival of smartphones, the ability to capture accurate locational information through a variety of applications (commonly referred to as ‘apps’) is now more common than ever because the smartphones have built-in GPS devices. Many social media apps (Twitter, Foursquare and Facebook are three common examples) have geo-location features that (optionally) includes the ability to attach locational information in the form of coordinates provided by the units GPS or place names provided by the user, thereby enabling individual users to leave a digital ‘geographic footprint’ of their movement when posting a message. This ‘geographical footprint’ is similar to that collected when a cell phone call or text message is sent. The major difference is that, unlike cell phone data, these records typically have precise GPS-generated locations rather than location to the nearest cell tower and can be freely collected independently of the software or service provider that generated the location data. Thus, the social activity with GPS coordinates can be aggregated to and summarized by various spatial/political units for different analytical purpose. Of the social media data, Twitter, through their geo-located tweets, has shown great potential for capturing human movement [18–21] across various political/regional boundary and is the focus of the research reported here.

Twitter has over 230 million active users [22] and its use is not confined to high income countries [20]. Twitter posts (tweets) are a maximum of 140 characters in length, but in spite of the brevity they carry a huge volume of publically available data within the average of 500 million tweets/day [22]. This data stream is attracting the attention of the scientific community as a source of information that may provide situational awareness about events (e.g. Senseplace2 [23]; ScatterBlogs [24]) as well as possible insights into society [25, 26]. Since 2010, Twitter provided users with the ability to include their location either by attaching coordinates or a place name [27] while tweeting, therefore making it possible to locate tweets geographically [18] both in space and over time (see [28]). The publicly accessible Streaming API provides around 90.1% coverage of the total geo-tagged tweets [29] and our own analysis of a 209 million tweet sample indicates that 91% of the tweets with geo-tagging are tagged with GPS-derived coordinates and 9% with place names (geolocated to common coordinates for that place). Geo-referenced tweets ordered in time by individual represent semi-continuous movement for that individual and because of the volume of tweets that are often sent, they can provide key insights into human movement patterns. Here we explored the utility of Twitter in capturing human movement in Kenya, where 62% of the population has access to a mobile phone (based on 2009 Census) [30]. The focus of our analysis was to understand movement patterns and connectivity between regions both nationally and internationally.

## Results

### Dataset summary

**Fig 1:**
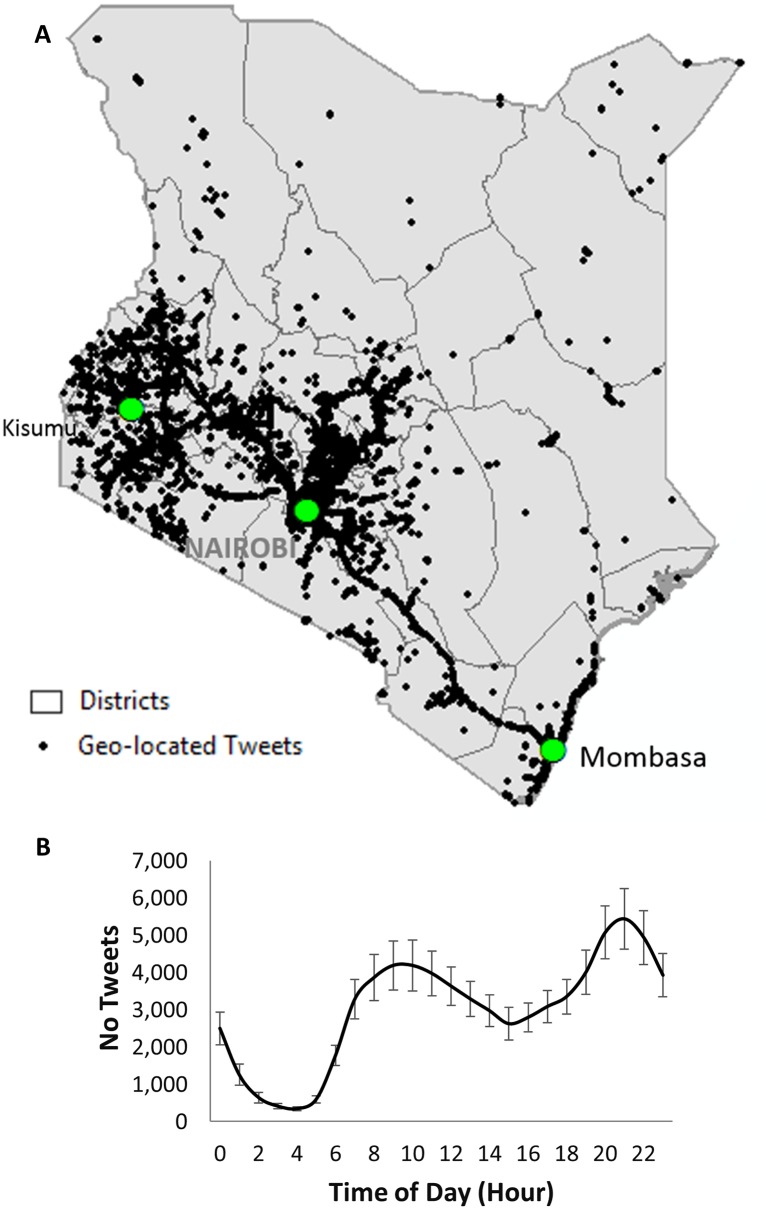
**(A)** Map showing distribution of tweets captured between June 2013 and March 2014. (B) Frequency of tweets per hour (+/- SE) local time. Map created using ArcGIS 10.2.

Geo-located tweets were captured over 10 months (June 2013 to March 2014) (Table 1) using the Twitter Streaming API version 1.1 (https://dev.twitter.com/docs/streaming-apis/streams/public) and saved to a text file in JSON format using a node.js application (http://nodejs.org/). To minimize erroneous movement and mobility results, we cleaned the data removed tweets that were related to web advertising (e.g. UNjobs_), traffic updates (e.g. NSC_MombasaRd) and internet bots (e.g. MarsBots). We further removed obvious errors in the data (e.g., users with apparent movement at speeds greater than 1000km/hr) [19]. A total of 720,149 tweets were captured for Kenya resulting in an average number of 4,931 tweets collected per day. Since we were only interested in movement we extracted latitude, longitude, date, time and the unique user id for each tweet. A total of 28,332 unique users,representing an estimated 1.2% of the population who have access to a mobile phone (N = 2.38 million [30]), were identified in Kenya (see Table 1 for summary) and mapped using ArcGIS 10.2 ESRI (Environmental Systems Research Institute). This percentage is very close to that found in a previous study [20]. Tweets were distributed throughout Kenya (Fig 1A) with the highest number of tweets captured in South Central and Western Kenya. Temporally at a daily scale, two peaks in the number of Tweets sent per day were apparent one, at 11am and the second at 9pm (Fig 1B). For the 10 months of data that were analyzed, tweets were highest during December and January and lowest in February (Table 1).

**Table 1:** Summary of Twitter data used in this analysis that was collected for Kenya between June 2013 and March 2014 (N_unique users_ = 28,335; N_tweets_ = 720,149).

Year	Date	No Unique Users	Total No Tweets per Month (Kenya)	Total No Tweets per Month (Kenya + surrounding countries)
**2013**	1 Jun– 30 Jun	6,405	88,377	135,845
	1Jul– 17 Jul	3,285	32,651	47,721
	3 Aug– 8 Aug	2,413	17,394	26,457
	1 Sep– 30 Sep	5,530	59,182	91,802
	1 Oct– 31 Oct	7,046	92,794	143,714
	1 Nov– 30 Nov	7,188	90,302	143,452
	1 Dec– 31 Dec	8,197	115,264	187,504
**2014**	1 Jan– 31 Jan	7,635	113,194	183,942
	1 Feb– 28 Feb	4,982	63,402	103,484
	1 Mar– 23 Mar	3,928	47,589	73,679

### Mobility and connectivity

We analyzed local and regional movement to identify how places are connected and demonstrate the utility of social media data for mapping connectivity. Each person creates a unique digital footprint with each message they send, therefore we ascertained movement patterns by connecting individual points by user. We constructed a series of temporal movement patterns. These included (i) daily movement patterns by connecting tweets for each user within a 24 hour time period; (ii) monthly movement patterns by joining tweets for each user for each month and (iii) total movement patterns by linking all tweets for each user. Movement tracks were created by connecting the tweet by date and time for each unique user. ESRI ArcGIS 10.2 was used to create movement tracks between each of the locations and calculate the distance between each tweet and the total distance travelled by each user.

**Fig 2:**
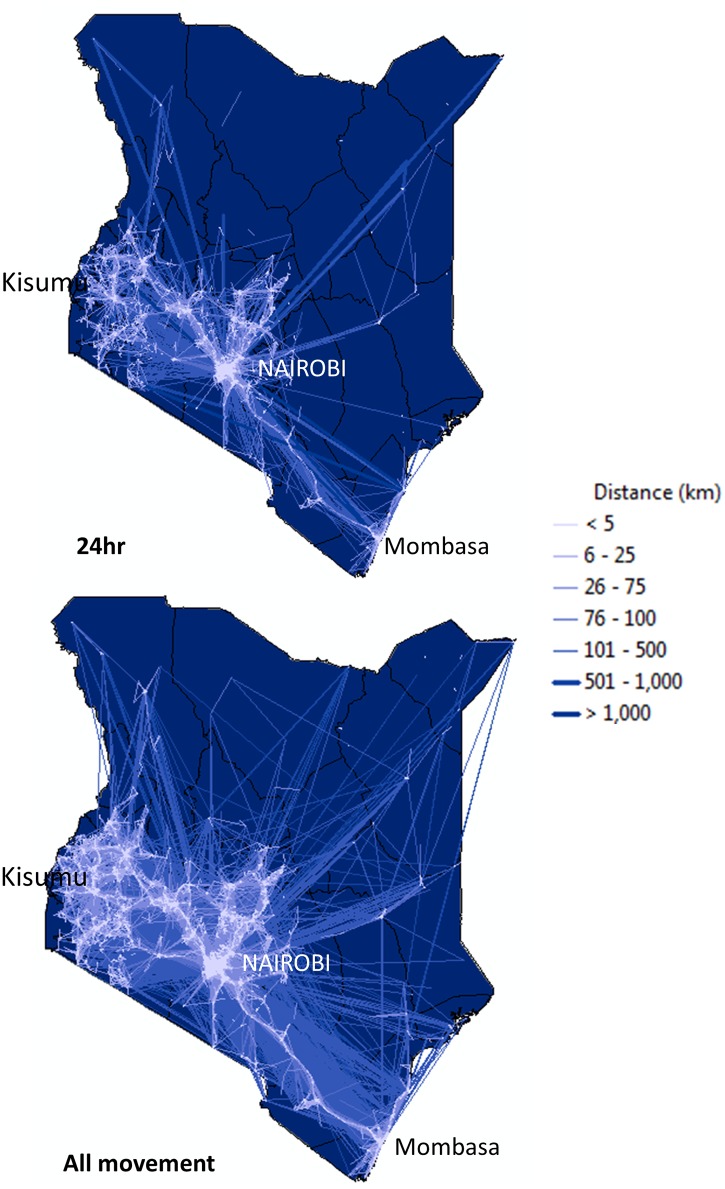
Movement patterns captured at different temporal scales illustrate connectivity between districts in Kenya within a 24-hour time period (N = 90,645 tracks) and during a ten month time period (N = 17,900). Each line represents a movement segment. The long distance tracks indicates population movements by plane or by train within the country. Maps created using ArcGIS 10.2.

**Fig 3:**
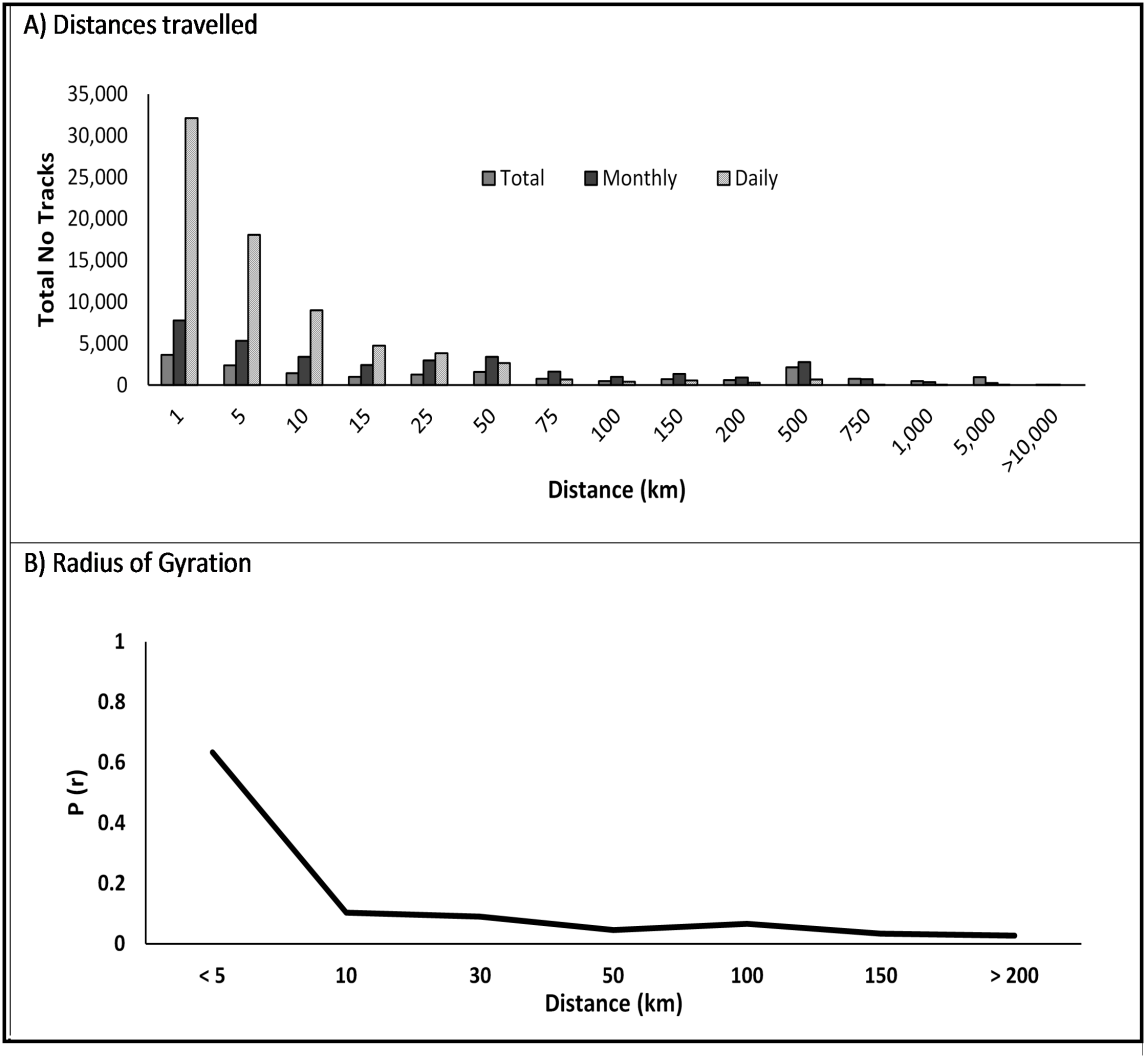
Connectivity between locations and human movement within Kenya. (A) Distances travelled daily, monthly and in total by each user and (B) the proportion of user’s radius of gyration (solid line).

Movement patterns captured at different temporal scales highlight changes in connectivity across Kenya (Fig 2). On average 427 movement tracks were recorded each day and much of this movement occurred over short distances (< 5km, Fig 3A). Estimates of the users’ spatial spread of movement based on analyzing their radius of gyration also indicated that the majority of users’ travelled mostly within a relatively small area (<5km, Fig 3B). As the temporal scale was expanded more long distance movement events were revealed, both within country and across national boundaries (Figs 2 and 3B).

### Net regional and international movement flow

**Fig 4:**
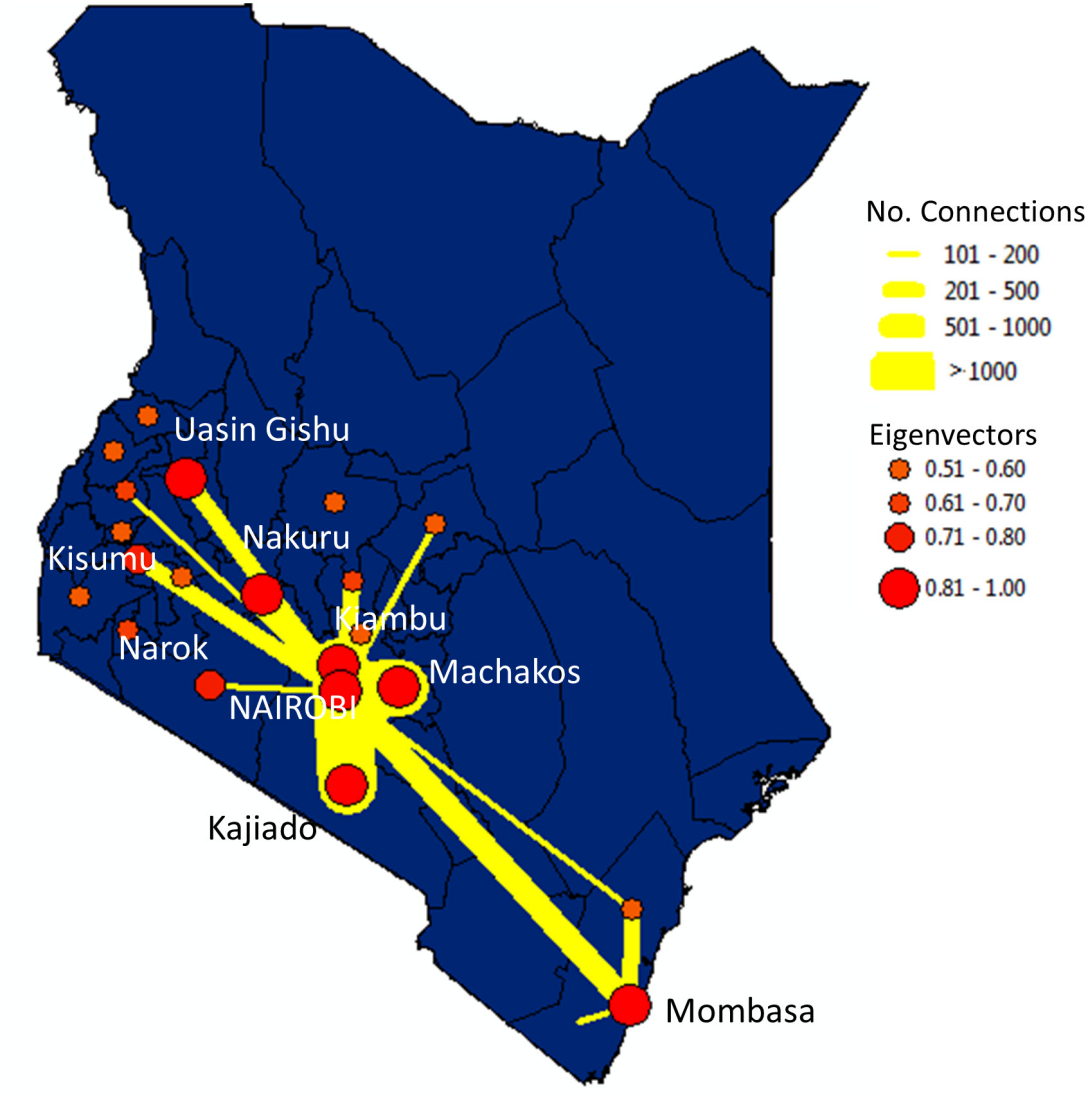
Map illustrating the connectivity between districts and Eigenvector value illustrating the level of influence of each district. For clarity we have only illustrated linkages with more than 100 connections and Eigenvectors greater than 0.50. Map created using ArcGIS 10.2.

Net regional flows of movement were constructed by capturing flow networks at administrative levels, where the administrative unit represents the source or destination nodes in each network. In this case we used centroids for district level boundaries as nodes in the network. Movement was defined as the movement of a person from one district to another. The total numbers of connections between districts were determined and used to investigate centrality of each district. We calculated four measures of centrality that included: degree (the number of ties to the district), betweenness (the number of times a district acts as a bridge between the shortest distance of two other districts), closeness (the inverse of the farness = the total distance of a district to all districts), and eigenvector centralities (the level of influence of a district by measuring the importance of a node based on the node’s connections) (Table 2). Fig 4 shows the districts with the highest connectivity. Matching the cell-phone based findings by [4], we also found that the capital, Nairobi, was a central hub in the travel network with movement flowing in and out of the capital to different parts of Kenya (Fig 4). Movement was highest between Nairobi and the neighboring districts Kiambu, Machakos and Kajiado in the Central Region. Additional strong connections were also identified between Nairobi and the coastal area of Mombasa and the districts in the west, Nakuru, Uasin Gishu and Kisumu (Table 2).

**Table 2:** Centrality values between districts in Kenya.

District Name	Degree	Betweenness	Closeness	Eigenvector
**BARINGO**	33	6.210	0.013	0.465
**BOMET**	33	2.908	0.013	0.469
**BUNGOMA**	40	11.894	0.014	0.539
**BUSIA**	33	3.581	0.013	0.467
**E. MARAKWET**	21	1.482	0.012	0.288
**EMBU**	34	8.217	0.013	0.450
**GARISSA**	29	13.653	0.013	0.347
**HOMA_BAY**	37	4.499	0.014	0.519
**ISIOLO**	28	7.912	0.013	0.327
**KAJIADO**	79	125.993	0.018	0.905
**KAKAMEGA**	49	14.293	0.015	0.653
**KERICHO**	43	18.557	0.013	0.569
**KIAMBU**	76	105.624	0.018	0.890
**KILIFI**	44	25.793	0.014	0.562
**KIRINYAGA**	36	6.346	0.013	0.497
**KISII**	44	10.389	0.014	0.606
**KISUMU**	59	39.176	0.016	0.735
**KITUI**	36	10.264	0.014	0.475
**KWALE**	36	7.276	0.013	0.491
**LAIKIPIA**	44	15.822	0.014	0.580
**LAMU**	13	0.148	0.011	0.174
**MACHAKOS**	72	74.364	0.018	0.866
**MAKUENI**	36	7.405	0.013	0.495
**MANDERA**	10	0.620	0.011	0.120
**MARSABIT**	10	0.250	0.011	0.133
**MERU**	47	23.142	0.014	0.590
**MIGORI**	36	7.067	0.013	0.490
**MOMBASA**	77	127.112	0.018	0.881
**MURANGA**	42	14.809	0.014	0.542
**NAIROBI**	94	266.909	0.022	1.000
**NAKURU**	77	98.524	0.018	0.908
**NANDI**	37	13.789	0.013	0.467
**NAROK**	59	44.211	0.016	0.742
**NITHI**	23	0.761	0.012	0.327
**NYAMIRA**	32	2.058	0.013	0.469
**NYANDARUA**	24	3.025	0.012	0.335
**NYERI**	49	19.072	0.015	0.628
**SAMBURU**	20	1.401	0.012	0.278
**SIAYA**	30	1.800	0.013	0.428
**TAITA TAVETA**	35	7.934	0.013	0.478
**TANA RIVER**	15	0.418	0.012	0.198
**TRANS-NZOIA**	38	8.474	0.014	0.522
**TURKANA**	21	2.106	0.012	0.291
**UASIN GISHU**	75	90.420	0.018	0.881
**VIHIGA**	42	7.944	0.014	0.585
**WAJIR**	15	1.760	0.012	0.197
**WEST POKOT**	21	1.588	0.012	0.299

### Cross-border movement and potential flow of parasite

**Fig 5:**
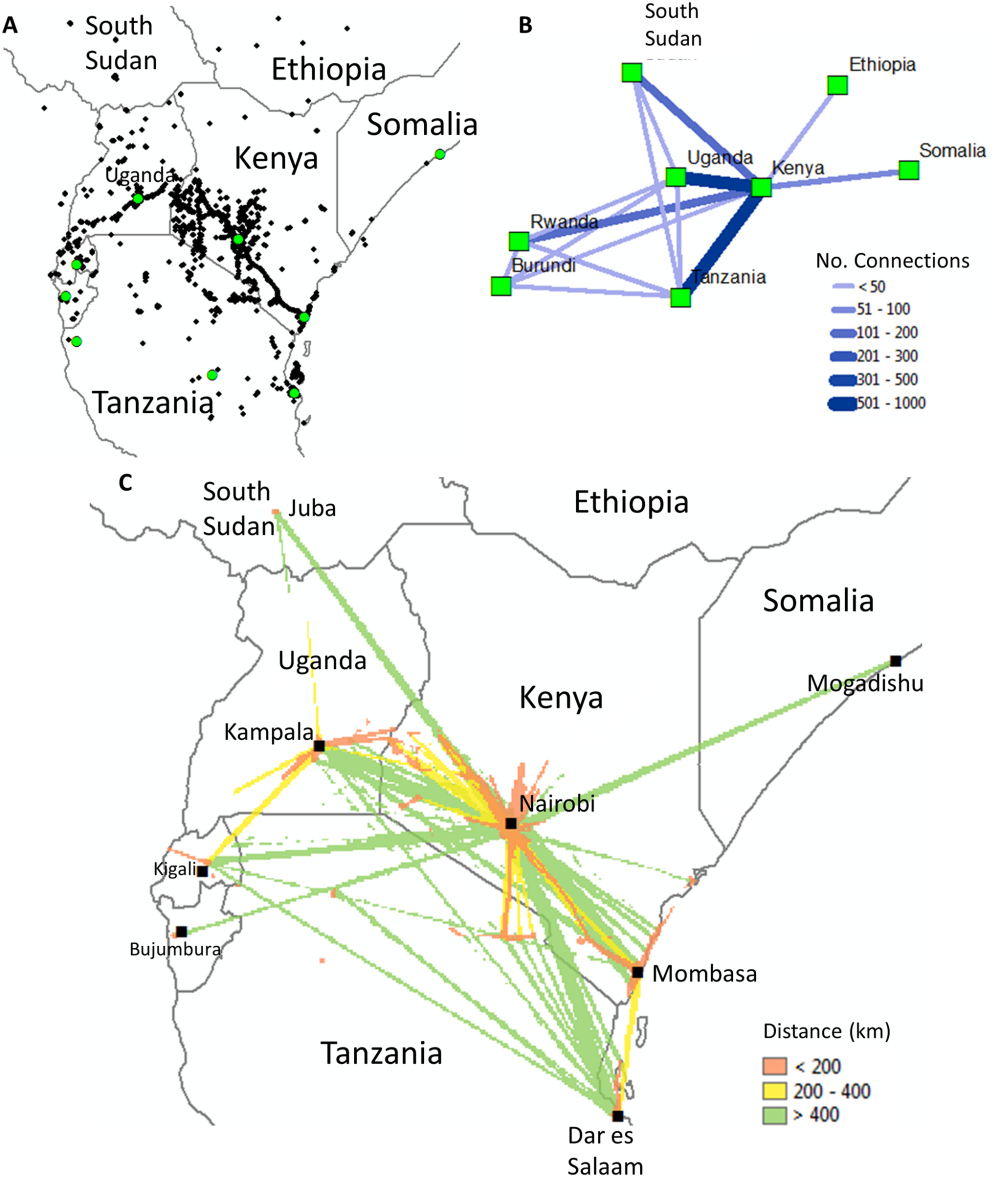
Maps illustrating (A) the distribution of geo-located tweets in the study area for users who crossed-borders (N = 770) (B) connections between Kenya and the surrounding countries and (C) a flow map showing the connectivity between different geographic locations by travel distance. Map created using ArcGIS 10.2.

A limitation of utilizing mobile phone data is that they do not capture cross-border movement since these data are restricted to the phone providers and coverage may not extend beyond country boundaries unless subscribers have the necessary roaming capabilities enabled. With Twitter, in contrast, unique user identifiers remain consistent, therefore users of social media sites can continue to log in to their accounts worldwide, provided they have access to the internet, and provide a way of capturing cross-border movement. We explored the ability to map cross-border movement by analyzing an additional 417,451 tweets (Table 1, Fig 5A). Approximately, three percent of the users who tweeted from Kenya also tweeted from the surrounding countries such as Uganda, Tanzania, Somalia and South Sudan (Fig 5A). Analysis of the movements recorded via Twitter found that eighty-five percent of these users only travelled between two countries, 11% between three countries, 2% between four countries and only two people visited five countries. Net flow of movement between countries is calculated using the centroids of each country as nodes in the network. Movement was defined as the movement of a person from one country to another and the total number of connections between each country was calculated. The greatest number of connections from and to Kenya were with Uganda and Tanzania (Fig 5B, darker and bolder the line the higher the number of connections). Since user’s movements are highly localized (Fig 3A), we further highlighted variations in long distance travel connections (green) versus more local connections that occur during shorter (brown) and intermediate (yellow) travel journeys (Fig 5C).

## Discussion

In this study we used geo-located Twitter data to determine movement flows in Kenya and the surrounding countries. Although Twitter data, like cell phone data, is limited due to the number of users tweeting and biases inherent in who has access to communication technology, these data can serve as a proxy for mobility [18–21] particularly in regions where data is scarce as shown here. Having perfect data is rare [31], yet in many cases the “good enough” principle in making decisions is sufficient [32]. Thus in absence of better data, Twitter may be considered a “good enough” data set for constructing human mobility networks and these data are likely to be more comprehensive than can be derived from the decennial census data, one-off surveys, or night lights methods.

Twitter has several advantages over mobile phone data records for tracking population movements: geo-located tweets provide greater locational accuracy than cell phone data since coordinate information is collected using built-in GPS receivers whereas cell phone data is linked to the nearest tower [18]. While this may not affect our ability to analyze large scale movement of people, the accuracy of small scale movement may be misrepresented when solely relying on cell phone data. Furthermore, cell phone data typically stops at country borders but Twitter data does not. A limitation of the study by [4] is that mobility was only quantified for Kenya. We have extended this study by capturing cross-border movement (Fig 5) and highlighted key connections between Kenya and surrounding countries (Fig 5B). For example, based on the number of connections shown in (Fig 5B), movement between Kenya and Tanzania is well established. By breaking this down further, we were able to distinguish travel patterns between specific geographic locations such as direct connections between Nairobi and Dar es Salaam and trips between Nairobi and Arusha (Figs 3 and 5C). Not only is this important for examining cross-border movement and importation of disease [33] but also for identifying disease epicenters by determining regional travel hubs that people pass through. For example, in light of the current Ebola outbreak, identifying major transport hubs, such as Nairobi in Kenya and their specific connections to other small as well as large locations at different times is important for determining places that may be at high risk to the virus [34]. Finally, the context of the tweets can reflect a population’s collected social behavior [35], which can be utilized to understand the spatial-temporal dynamics of peer influence and social contagion of behavioral spread [36].

**Fig 6:**
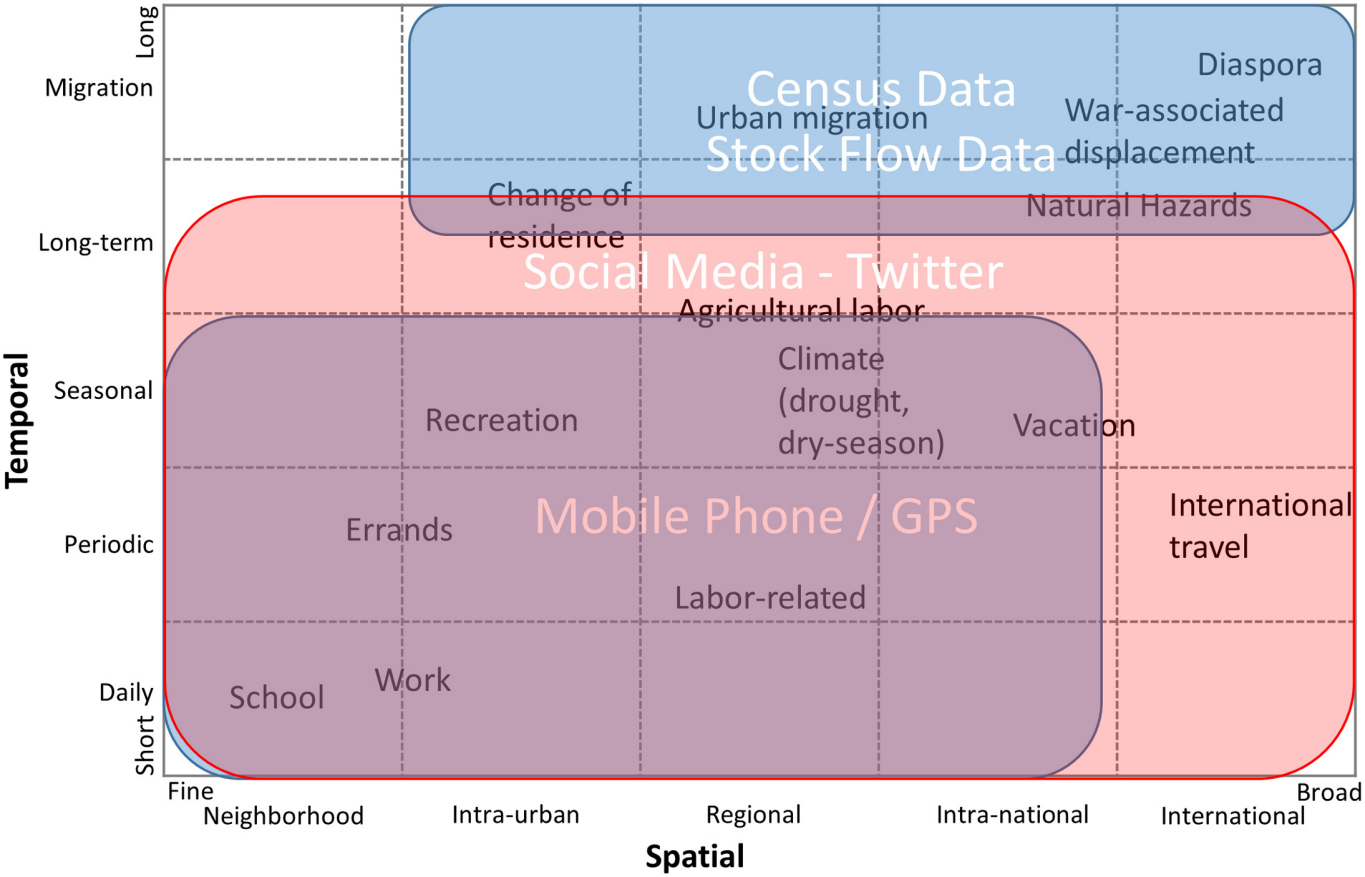
A framework and associated data sources useful for capturing human mobility in time and space. Movements are characterized in terms of their spatial and temporal scale, which are defined in terms of physical displacement (*spatial*) and time spent (*temporal*, frequency and duration) (Source: adapted from [45, 57]).

Movement can take place for many reasons ranging from work and economic well-being [1] through conflict [37] to displacement caused by loss of livelihoods due to natural hazards (e.g. climate- and weather-related events [38] such as flooding [39, 40], drought [41, 42],[14] and heat stress [43]) at different temporal (e.g. daily to migration) and spatial scales (neighborhood, city-to-city, to international). Twitter has the potential to capture human movement across these different spatial and temporal scales as highlighted by the red box in (Fig 6). Also, with continuous growth and wide-spread user distribution, geotagged tweets show great potential to serve as a complement to cell phone based data [15] for population distribution estimation [44].

Twitter is free currently, easily accessible and can provide on-the-ground information at local, national and international levels. The key contributions of the study presented here are demonstrating the usefulness of Twitter for mapping human movement and, for Kenya, highlighting key movement corridors and convergence zones that may serve as social hotspots. Having the ability to pinpoint these areas will be useful for policy development and planning, particularly with limited public resources [45]. Although this study examined the utility of Twitter for delineating human movement patterns, the usefulness of this type of data could easily be extended to other applications including, but not limited to, improve disease modelling [9], understand spatial/temporal epidemic dynamics and disease movements [16], enhance disease surveillance [46], understanding of community structure [47, 48], delineating people’s activity space [49], assessing access to healthcare or vaccination centers [50] and understanding people’s movement response during an emergency (e.g. Hurricane Sandy [51, 52]). Therefore the work outlined here provides a lens for examining the complexities and heterogeneities of movement in both space and time that enables us to gain further insights into the motivations associated with mobility patterns in different settings such as data-rich and data-poor countries and urban vs rural settings.

## Methods

### Mobility measures

The mobility of an individual was measured using the radius of gyration trajectory, *r(g)* where the location of an individual, *i*, at a location in space and time is given by the geolocation of that individual’s tweet. Since we do not know an individual’s home location, the center of mass, or centroid of all known user locations, was used to represent the user’s usual location and was calculated as:

$$m_{cm}=\frac{1}{n}\sum_{i=1}^{n}m_{i}$$ (1)

where *m*_*i*_ is the location of a geo-located tweet during the time period for which data was collected.

For each user the radius of gyration was calculated:

$$r(g)=\sqrt{\frac{1}{n}\sum_{i=1}^{n}{\left|m_{i}-m_{cm}\;\right|}^{2}}$$ (2)

where *n* is the number of locations from where a tweet was sent, *m*_*i*_ is the location of a tweet and *m*_*cm*_ is the center of mass of a trajectory [6].

### Centrality calculations

Firstly, we calculated the degree of a node as the number of edges it is connected to. This is the first implication of how districts are connected by population movements. Afterward, we calculated the betweenness centrality, closeness centrality and eigenvector centrality. Betweenness centrality calculates the number of shortest paths going through a specific vertex in a network. In transport network analysis, it provides an approximating measure of the traffic handled by the vertices, as well as an indicator of the importance of the vertices in a network [53]. The betweenness centrality is calculated as follows:

$$C_{B}(v)=\sum_{s\neq v\neq t}\frac{\delta_{st}(v)}{\delta_{st}}$$ (3)

In which *δ*_*st*_ is the total number of shortest paths from node s to node t and *δ*_*st*_(*v*) is the number of those paths that pass through v. Under our population movement network context, the vertices betweenness centrality can be interpreted as the intensity that population movement going through vertices.

Closeness centrality measures the distance from a node to all other nodes in a network [54]. It is calculated based on the length of the average shortest path between a vertex and all vertices in the graph:

$$C_{C}(n_{i})={\left[\sum_{j=1}^{g}d(n_{i},n_{j})\right]}^{-1}$$ (4)

In which *d*(*n*_*i*_, *n*_*j*_) is the distance between two nodes. In our population movement network setting, the closeness centrality can be interpreted as how close the districts are connected by population movement.

Eigenvector centrality measures relative importance of a node in a network [55, 56]. If a node has more connections to highly connected nodes, it has a relatively higher score than for one that has more connections to less connected nodes. The eigenvector centrality is calculated as follows:

$$x=\frac{1}{\lambda }Ax,\;x_{i}=\frac{1}{\lambda }\sum_{j=1}^{n}a_{ij}x_{j}$$ (5)

in which A would be the adjacency matrix, *λ* is the largest eigenvalue and x is the corresponding eigenvector. Spatial clusters of eigenvector centrality can be observed on (Fig 4), which can be interpreted as high volume of population movements vertices are close to each other in geographic distance.
